# The Role of Forced and Voluntary Training on Accumulation of Neural Cell Adhesion Molecule and Polysialic Acid in Muscle of Mice with Experimental Autoimmune Encephalomyelitis

**DOI:** 10.1155/2020/5160958

**Published:** 2020-04-08

**Authors:** Farzaneh Torabimehr, Mohammad Reza Kordi, Reza Nouri, Jafar Ai, Sadegh Shirian

**Affiliations:** ^1^Department of Neuromuscular Exercise Physiology, Kish International Campus, Tehran University, Kish Island, Iran; ^2^Department of Exercise Physiology, University of Tehran, Tehran, Iran; ^3^Department of Tissue Engineering and Applied Cell Sciences, School of Advanced Technologies in Medicine, Tehran University of Medical Sciences, Tehran, Iran; ^4^Department of Pathology, School of Veterinary Medicine, Shahrekord University, Shahrekord, Iran; ^5^Shiraz Molecular Pathology Research Center, Dr Daneshbod Pathol Lab, Shiraz, Iran; ^6^Biotechnology Research Institute, Shahrekord University, Shahrekord, Iran; ^7^Shefa Khatam Neuroscience Research Center, Tehran, Iran

## Abstract

It has been suggested that depletion of adhesion molecules is one of the factors associated with or possibly responsible for multiple sclerosis (MS) progression. The aim of this study was to investigate the effect of forced and voluntary training before and after induction of experimental autoimmune encephalomyelitis (EAE) on accumulation of neural cell adhesion molecule (NCAM) and polysialic acid (PSA) in neuromuscular junction denervation in plantaris and soleus muscles in C57BL/6 female mice. A total of 40 female C57BL/6 mice, 10-week-old, were randomly divided into four groups, including induced control groups without EAE induction, induced EAE without training, and forced and voluntary training groups. Myelin oligodendrocyte glycoprotein peptide 35–55 (300 *μ*g in saline; MOG 35–55; KJ Ross-Petersen ApS, Denmark) was injected subcutaneously at the base of the tail of each mouse. Clinical assessment of EAE was performed daily using a 15-point scoring system following immunization. Training groups performed the swimming program for 30 min/day, 5 times/week, for 4 weeks. Mice had access to a treadmill for one hour per day, 5times/week, for 4 weeks in individual cage. The mice were scarified, and the plantaris and soleus muscles were then isolated for investigation of proteins expression using IHC. An analysis of the preventive exercise (before) and recovery exercise (after) of the EAE was performed. Images of the stained sections were taken using a fluorescent microscope. Quantitative image analysis was performed using ImageJ software package. The obtained data from the mean percentage expression of PSA and NCAM in pre- and post-soleus and plantaris muscles showed that the highest and lowest expression levels of PSA and NCAM belonged to control and swim EAE (SE) groups, respectively. The low expression levels of PSA and NCAM were detected in rat with MS without intervention. In conclusion, the relationship between increasing levels of NCAM and PSA protein expression and voluntary and compulsory activity were detectable both in pre and post-soleus and plantaris. However, voluntary activity resulted in more expression levels of NCAM and PSA than that of compulsory. In conclusion, since it has been suggested that depletion of NCAM is one of the factors associated with or possibly responsible for MS progression, these findings show exercise MS progression may be reduced by increasing expression of exercise-related adhesion molecule such as NCAM and PSA (a glycan modification of the NCAM).

## 1. Introduction

Multiple sclerosis (MS) is a chronic complex T cell-mediated autoimmune disease of the central nervous system (CNS). It is characterized by demyelination, axonal degeneration, and neuronal inflammation with a prominent neurodegenerative component [1]. In MS, several adhesion molecules are involved within the CNS in inflammatory and neurodegenerative processes [2]. It has been suggested that depletion of neural cell adhesion molecule (NCAM) is one of the factors associated with or possibly responsible for MS progression [3]. The NCAM, a member of the immunoglobulin superfamily, is expressed on both neurons and glial cells that has been intimately linked to axonal outgrowth, remyelination processes, guidance, and fasciculation and participate in the reparative mechanisms [4]. Isoforms of the NCAM carrying the linear homopolymer of alpha 2, 8-linked polysialic acid (PSA) also can provide unique properties in neural migration [4, 5]. Therefore, NCAM-PSA appears to play a critical role in mediating precursor cell migration in the brain [6]. PSA is a glycan modification of the NCAM produced by the polysialyl transferases ST8SIA2 and ST8SIA4. PSA has been detected in MS plaques, but its adverse or beneficial role in remyelination is controversial. It has been recently shown that NCAM and ST8SIA2 promote oligodendrocyte differentiation and myelin repair, while ST8SIA4 delays oligodendrocyte differentiation, explaining its adverse role in remyelination [7].

It has also been suggested that not only regular exercise may benefit cognitive function by rescuing some hippocampal cellular and molecular impairments but also it has a direct neuroprotective effect in EAE, an animal model of MS. Several molecules such as NCAM and PAX7 are commonly used for detecting myogenic precursor cells and identifying their activation status. Caldow et al. [8] have demonstrated that the expression of the NCAM increase following acute resistance exercise to indicate a rapid initiation of the myofiber repair process. NCAM is expressed during myoblast proliferation and maturation [9], while PAX7 is expressed during quiescence and activation of myogenic precursor cells [10]. The NCAM and its PSA moiety are also essential for proper motor axon guidance in the developing neuromuscular system [11]. In MS patients, there is impaired balance and gait due to the process of demyelination as well as identified certain symptoms of motor disorders. These disorders can be estimated by quantifying neuromuscular and cortical parameters [12]. Here, we investigated the effect of forced and voluntary training pre- and postinduction of EAE on accumulation of NCA and PSA in neuromuscular junction denervation in plantaris and soleus muscles in C57BL/6 female mice.

## 2. Materials and Methods

A total of 40 female C57BL/6 mice, 10-week-old, were randomly divided into four groups, including induced control groups without EAE induction, induced EAE without training, and forced and voluntary training groups. All animals have started training 4 weeks before experimental autoimmune encephalitis- (EAE-) induction. The mice were intraperitoneally anesthetized with an injection of xylazine (5 mg/kg) and ketamine (50 mg/kg). Myelin oligodendrocyte glycoprotein peptide 35–55 (300 *μ*g in saline; MOG 35–55; KJ Ross-Petersen ApS, Denmark) was emulsified in an equal volume of complete Freund's adjuvant (CFA; Sigma, USA) containing 500 *μ*g of heatkilled *Mycobacterium tuberculosis*. This emulsion was then injected subcutaneously at the base of the tail of each mouse. The mice also received intraperitoneal injection of pertussis toxin (300 ng in 10 *μ*l PBS; List Biological Lab, USA) at the time of immunization and the same was repeated 48 hours later. To confirm EAE induction, animal clinical assessment of EAE was performed daily using a 15-point scoring system following immunization as reported previously by Giuliani et al. [13].

### 2.1. Compulsory Activity

Training groups performed the swimming program for 30 min/day, 5 times/week, for 4 weeks.

Mice were monitored during exercise sessions and encouraged to continue using a sponge. The exercise protocol was the same pre- and posttraining.

### 2.2. Voluntary Activity

Mice had access to a treadmill for one hour per day, 5 times/week, for 4 weeks in individual cage. The mileage was recorded using a computer. The mice were euthanized, and the plantaris and soleus muscles were then isolated for investigation of proteins expression using IHC.

### 2.3. IHC Staining

Localization of NCAM and PSA in plantaris and soleus muscles was studied using IHC. Tissue samples were fixed in 10% natural buffered formalin embedded in paraffin. Paraffin sample sections with a thickness of 10 *µ*m were used for IHC analysis as previously reported by Ghavideldarestani et al. [14]. In brief, the tissue slides were deparaffinized on an incubator for 1 hour and washed twice with xylene, each time for 10 minutes. The sections were incubated with blocking solution containing 4% goat serum for 30 min in PBS to prevent nonspecific staining by placing the slides in a humidifier chamber. The sections were then treated with 5% normal donkey serum (NDS; Jackson Laboratories) diluted in PBS with 0.2% Triton-X100 (Sigma) for 1h and were incubated overnight with anti-PSA, NCAM, and PSA-NCAM mouse monoclonal antibody diluted (Abcam, USA) at 1 : 500 in 0.1 M PBS containing 1% (w/v) bovine serum albumin (BSA) and 1% (v/v) normal goat serum in a dark sealed container at room temperature. The sections finally were exposed to fluorescein isothiocyanate- (FITC-) conjugated goat anti-mouse IgM (Abcam, UK) diluted 1 : 200 for 3 h. All sections were costained with *α*-bungarotoxin (Abcam, USA) (1 : 100, Sigma-Aldrich, USA) to identify the neuromuscular junction. The slides were mounted with 4′, 6-diamidino-2-phenylindole (DAPI) (Vectashield mountingmedium with DAPI, Vector Laboratories, California, USA).

### 2.4. Quantitative Evaluation of PSA-NCAM Expression

Images of the stained sections were taken using a fluorescent microscope. Quantitative image analysis was performed using ImageJ software package.

### 2.5. Statistical Analysis

The data were analyzed by SPSS version 22. Differences between the mean expressions of NCAM and PSA in various groups were analyzed by one-way ANOVA. Effects were considered significant at *p* <  0.05. Data are presented as the mean ± SD.

## 3. Results

**Figure 1:**
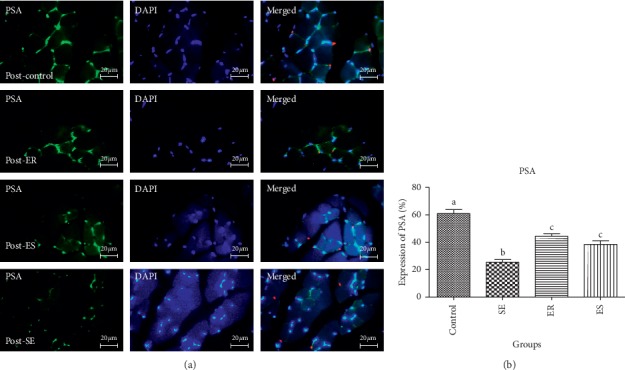
The expression of PSA post-soleus muscle. The mean percentage of PSA expression in control group was significantly higher than those of compared to SE (*P* < 0.0001), ER (*P* < 0.0001), ES (*P* < 0.0001) groups. The mean percentage expression of PSA in post-soleus was significantly down-regulated in SE group compared to ER (*P* < 0.0001) and ES (*P* < 0.0001) groups. There was significant difference in the mean percentage expression of PSA of post-soleus muscle in ER group compared to ES group.

**Figure 2:**
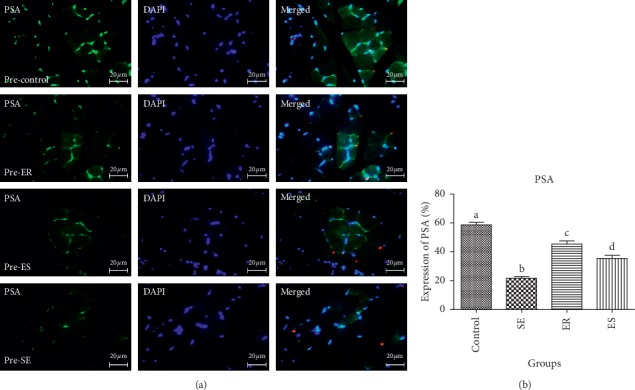
The expression of PSA pre-soleus muscle. The mean percentage of PSA expression in control group was significantly higher than those of compared to SE (*P* < 0.0001), ER (*P* < 0.0001), ES (*P* < 0.0001) groups. The mean percentage expression of PSA in post-soleus was significantly down-regulated in SE group compared to ER (*P* < 0.0001) and ES (*P* < 0.0001) groups.

The obtained data from the mean percentage expression of PSA in pre- and post-soleus muscle showed that the control group (58.71 ± 3.20%, 60.97 ± 5.10%, respectively) showed significantly higher mean percentage expression of PSA compared to SE (21.73 ± 2.24%, *P* < 0.0001; 25.50 ± 3.34%, *P* < 0.0001, respectively), ER (45.41 ± 3.81%, *P* < 0.0001; 44.46 ± 3.22%, *P* < 0.0001, respectively), and ES (35.40 ± 3.73%, *P* < 0.0001, 38.50 ± 4.49%; *P* < 0.0001, respectively) groups (Figures 1 and 2). The mean percentage expression of PSA in pre- and post-soleus was significantly downregulated in the SE group compared with that of ER (*P* < 0.0001) and ES (*P* < 0.0001) groups (Figure 1). Unlike pre-soleus muscle, there was significant difference in the mean percentage expression of PSA of post-soleus muscle in the ER group compared with that in the ES group (Figures 1 and 2).

The mean percentage expression of NCAM in pre- and post-soleus muscle showed that the control group (61.36 ± 2.56%, 64.96 ± 2.03%, respectively) expressed significantly higher percentage expression of NCAM than that in SE (22.46 ± 1.71%, *P* < 0.0001; 25.87 ± 3.69%, *P* < 0.0001, respectively), ER (51.92 ± 1.92%, *P* < 0.0001; 49.41 ± 2.81%, *P* < 0.0001, respectively), and ES (39.13 ± 2.48%, *P* < 0.0001; 46.07 ± 2.91%, *P* < 0.0001, respectively).

**Figure 3:**
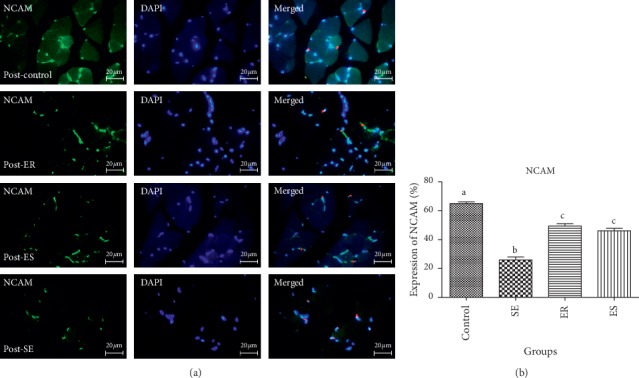
The expression of NCAM in post-soleus muscle. The mean percentage of NCAM expression in control group was significantly higher than those of compared to SE (*P* < 0.0001), ER (*P* < 0.0001), ES (*P* < 0.0001) groups. The mean percentage expression of NCAM in post-soleus was significantly down-regulated in SE group compared to ER (*P* < 0.0001) and ES (*P* < 0.0001) groups.

**Figure 4:**
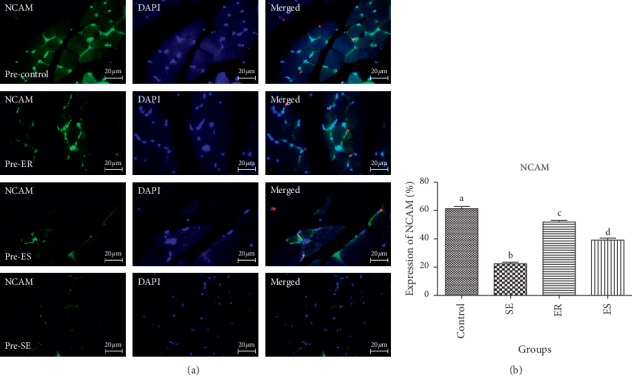
The expression of NCAM in pre-soleus muscle. The mean percentage of NCAM expression in control group was significantly higher than those of compared to SE (*P* < 0.0001), ER (*P* < 0.0001), ES (*P* < 0.0001) groups. The mean percentage expression of NCAM in pre-soleous was significantly down-regulated in SE group compared to ER (*P* < 0.0001) and ES (*P* < 0.0001) groups.

The mean percentage expression of NCAM in pre- and post-soleus was significantly downregulated in the SE group compared with that in ER (*P* < 0.0001) and ES (*P* < 0.0001) groups (Figure 2). However, there was no significant difference in the mean percentage expression of NCAM of pre- and post-soleus muscle in the SE group compared with that in ER and ES groups (Figures 3 and 4).

**Figure 5:**
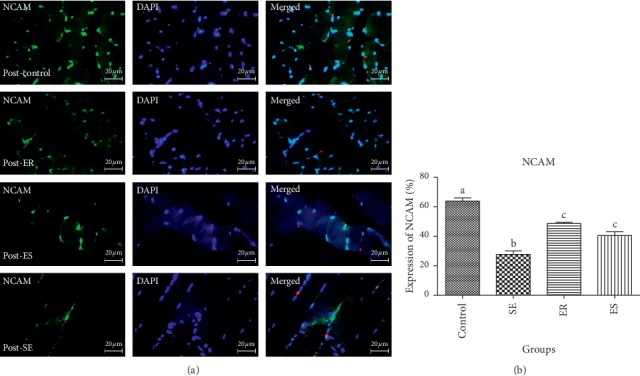
In post-EDL, the mean percentage of NCAM expression in control group showed significant increase in control group compared to SE (*P* < 0.0001), ER (*P* < 0.0001), ES (*P* < 0.0001) groups. The mean percentage expression of NCAM in post-EDL was significantly down-regulated in SE group compared to ER (*P* < 0.0001) and ES (*P* < 0.0001) groups.

**Figure 6:**
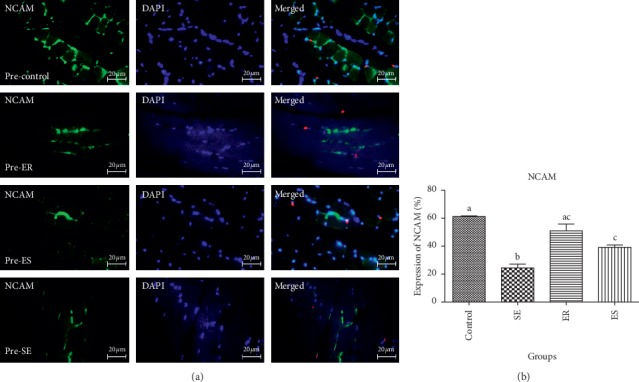
In pre-EDL, the mean percentage of NCAM expression in control group showed significant increase in control group compared to SE (*P* < 0.0001), ER (*P* < 0.0001), ES (*P* < 0.0001) groups. The mean percentage expression of NCAM in post-EDL was significantly down-regulated in SE group compared to ER (*P* < 0.0001) and ES (*P* < 0.0001) groups.

In pre- and post-EDL, the mean percentage of NCAM expression in the control group showed significant increase in the control group (61.24 ± 0.78, 63.82 ± 3.71%, respectively) compared with that in SE (24.43 ± 4.75, *P* < 0.0001; 27.66 ± 4.22%, *P* < 0.0001%, respectively), ER (51 ± 8.43, *P* < 0.0001; 48.61 ± 1.59%, *P* < 0.0001%, respectively), and ES (39.23 ± 2.90, *P* < 0.0001; 40.47 ± 4.45%, *P* < 0.0001) groups (Figure 3). The mean percentage expression of NCAM in pre- and post-EDL was significantly downregulated in the SE group compared with that in ER (*P* < 0.0001) and ES (*P* < 0.0001) groups. There was no significant difference in the mean percentage expression of NCAM of pre- and post-EDL in the SE group compared with that in ER and ES groups (Figures 5 and 6).

**Figure 7:**
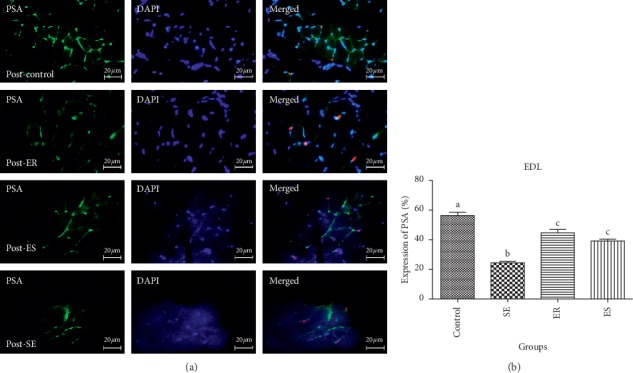
In post-EDL, the mean percentage of PSA expression in control group showed significant increase in control group compared to SE (*P* < 0.0001), ER (*P* < 0.0001), ES (41 ± 2.16, *P* < 0.0001) groups. The mean percentage expression of PSA in post-EDL was significantly down-regulated in SE group compared to ER (*P* < 0.0001) and ES (*P* < 0.0001) groups.

**Figure 8:**
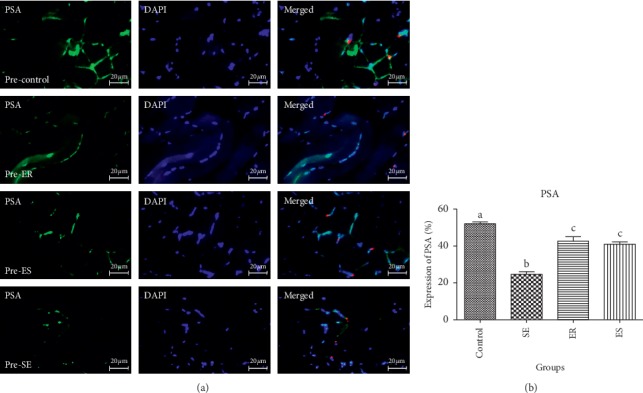
In pre-EDL, the mean percentage of PSA expression in control group showed significant increase in control group compared to SE (*P* < 0.0001), ER (*P* < 0.0001), ES (41 ± 2.16, *P* < 0.0001) groups. The mean percentage expression of PSA in pre-EDL was significantly down-regulated in SE group compared to ER (*P* < 0.0001) and ES (*P* < 0.0001) groups.

In pre- and post-EDL, the mean percentage of PSA expression in the control group showed significant increase in the control group (52.06 ± 1.78, 56.27 ± 3.81%, respectively) compared with that in SE (24.63 ± 2.53, *P* < 0.0001; 24.41 ± 1.75%, *P* < 0.0001%, respectively), ER (42.62 ± 4.29, *P* < 0.0001; 44.63 ± 4.06%, *P* < 0.0001%, respectively), and ES (41 ± 2.16, *P* < 0.0001; 39.07 ± 2.19%, *P* < 0.0001) groups (Figure 3). The mean percentage expression of PSA in pre- and post-EDL was significantly downregulated in the SE group compared with that in ER (*P* < 0.0001) and ES (*P* < 0.0001) groups. There was no significant difference in the mean percentage expression of NCAM of pre- and post-EDL in the SE group compared with that in ER and ES groups (Figures 7 and 8).

## 4. Discussion

The aim of this study was to investigate the effect of forced and voluntary training pre- and postinduction of EAE on accumulation of NCAM and PSA in neuromuscular junction denervation in plantaris and soleus muscles in C57BL/6 female mice. A growing body of evidence has implicated NCAM and PSA as risk factors for major neuropsychiatric and neurodegenerative disorders [15]. The obtained data from the mean percentage expression of PSA and NCAM in pre- and post-soleus and plantaris muscles showed that the highest and lowest expression levels of PSA and NCAM belonged to the control and SE groups, respectively. The low expression levels of PSA and NCAM were detected in rat with MS without intervention. The PSA expression results suggest that compulsory and voluntary exercise can increase PSA and NCAM protein expression of Spre- and post-soleus and plantaris muscles in EAE animals. Moreover, voluntary wheel running exercise significantly increased PSA and NCAM protein expression compared with compulsory exercise in post-soleus muscle. However, the benefits of exercise in the expression of PSA and NCAM proteins in pre- and post-soleus and plantaris muscles in EAE animals are demonstrated in this study. Adhesion molecules such as PSA and NCAM are suggested to contribute in the different processes that result in the progress of lesions and neurodegeneration in MS [2, 7]. PSA is a glycan modification of the NCAM that has been detected in MS plaques, but its beneficial or adverse role in remyelination is controversial [7].

It has been suggested that PSA-NCAM may modulate the functional interaction between neurotrophin brain-derived neurotrophic factor (BDNF) and its high and low affinity receptors. So, the distribution of the BDNF and PSA-NCAM modulates BDNF signaling [16]. It has been recently shown that the forced high intensity exercise may diminish the protein concentration of BDNF in soleus muscle [17].

The endoplasmic reticulum (ER) modulates various physiological processes including protein folding and calcium homeostasis to maintain cellular homeostasis [18]. Multiple pathologic factors, such as oxidative stress triggering prolonged disorders or distress on ER homeostasis, resulting in the unfolded protein response pathway in the ER lumen is known as ER stress [19]. Changes in the inflammatory profile and inhibition of immune responses induced by exercise cause alterations in the oxidative stress parameters [20]. Molecular adhesion molecules such as NCAM protein is synthesized in the ER as a membrane-bound glycoprotein that can undergo various posttranslational modifications before reaching the cell surface. Collectively, the ameliorative effect of exercise on ER stress-associated risk factors such as inflammation and oxidative stress can be important in the expression of molecular adhesion [21]. Therefore, the low expression of NCAM and PSA in EAE mice may attribute to the effect of inflammation and stress oxidative activity in neuromuscular junction as well as axonal damage. In contrast, the higher expression level of these proteins in voluntary and compulsory activity mice groups may attribute to the ameliorative effect of exercise [19]. The reductions in autoimmune cell infiltration, delay in onset of clinical disability, attenuation of severity, and preservation of axons and motor neurons in the lumbar spinal cord of mice with EAE with voluntary wheel running have been previously reported [22]. Exercise has also been associated with preserving or enhancing of physical function in human with MS, indicating the neuroprotection role of exercise [23]. It has been shown that the proteins deterioration occurs directly or indirectly following oxidative stress including damage to specific amino acid residues, peroxidation, changes, or degradation in their tertiary structure and lose of enzymatic activity [24]. These changes lead to alterations in the type and level of cellular proteins especially NCAM as well as physiological cellular functions [24]. The association of immunomodulatory effects of exercise with the expression of tight-junction proteins and reduced adhesion molecules expression, especially of extracellular vascular cell adhesion molecule 1 (VCAM-1), platelet and endothelial cell adhesion molecule 1 (PECAM-1), and intercellular adhesion molecules 1 (ICAM-1) have been previously reported [25, 26]. It has been recently shown that the biological pathways linking these adhesion molecules alter in MS [2]. Cell adhesion molecules such as NCAM play an important role in signal transduction cell, migration and proliferation, neurite outgrowth and fasciculation, and synaptogenesis and synaptic plasticity [27]. NCAM promotes and regulates synaptic stability and strongly influences neurotransmission. NCAM functions depend on its glycosylation, particularly on polysialylation, i.e., attachment of PSA [28]. The role of NCAM and its associated PSA as an important mediator of synaptic plasticity has been widely recognized [28]. It has been demonstrated that PSA gene and protein expression level are regulated in an age-, cell type-, and activity-dependent manner [15]. Spasticity, perceived by patients as muscle rigidity and spasms, is a common symptom in MS [29] that may be reduced by exercise.

In conclusion, the relationship between increasing levels of NCAM and PSA protein expression and voluntary and compulsory activities was detectable both in pre- and post-soleus and plantaris. However, voluntary activity resulted in more expression levels of NCAM and PSA than those of the compulsory one. In conclusion, since it has been suggested that depletion of NCAM is one of the factors associated with or possibly responsible for MS progression, these findings show exercise MS progression may be reduced by increasing expression of exercise-related adhesion molecule such as NCAM and PSA (a glycan modification of the NCAM).

## Data Availability

All data used to support the findings of this study can be made available from the corresponding author upon request (mrkordi@ut.ac.ir).
